# Identification and Quantitation of Bioactive and Taste-Related Dipeptides in Low-Salt Dry-Cured Ham

**DOI:** 10.3390/ijms23052507

**Published:** 2022-02-24

**Authors:** Alejandro Heres, Marta Gallego, Leticia Mora, Fidel Toldrá

**Affiliations:** 1 Instituto de Agroquímica y Tecnología de Alimentos (CSIC), Avenue Agustín Escardino 7, 46980 Paterna, Spain; alejandergo@iata.csic.es (A.H.); ftoldra@iata.csic.es (F.T.); 2 Departamento de Tecnología de Alimentos, Universitat Politècnica de València, Camino de Vera s/n, 46022 Valencia, Spain; margalib@upv.es

**Keywords:** mass spectrometry, triple quadrupole, bioactivity, peptidomics, peptides, curing, meat products, dry-cured ham

## Abstract

The reduction of salt in meat products influences the natural mechanisms of proteolysis occurring in their processing, and could affect the final characteristics of the product in terms of texture and flavor due to its effect on the activity of enzymes. In the present study, the quantitation of dipeptides PA, GA, VG, EE, ES, DA, and DG in low-salt Spanish dry-cured ham was carried out using a triple quadrupole mass spectrometry instrument. The developed methodology demonstrated the advantages of hydrophilic interaction liquid chromatography in the removal of salt as a clean-up/separation step before ionization. This resulted in a value of 44.88 μg/g dry-cured ham for GA dipeptide, and values ranging from 2 to 8 μg/g dry-cured ham for VG, EE, ES, DA, and DG dipeptides. PA showed the lowest concentration with a value of 0.18 μg/g dry-cured ham. These outcomes prove the remarkable activity of muscular dipeptidyl peptidases during dry-curing as well as confirming the presence of these dipeptides which are related to certain taste attributes (e.g., ‘bitter’ or ‘umami’). Such dipeptides have also been confirmed as anti-inflammatory and potential cardiovascular protectors using in vitro assays, with the advantage of dipeptides small size increases their chance to resist both gastrointestinal digestion and intestinal/bloodstream transport without being degraded or modified.

## 1. Introduction

Sodium chloride has been traditionally used in dry-cured meat products as an ingredient to control its safety by reducing water activity and controlling microbial spoilage. Salt has also been used to improve the sensory characteristics of the final product, contributing to a flavor enhancing effect and influencing the generation of flavor-related compounds in meat products [1]. Salting is an essential step in the production of dry-cured ham. In this sense, the use of salt in meat products also influences the natural mechanisms of proteolysis and lipolysis that occur during their processing by affecting the final characteristics of the product in terms of texture and flavor [2].

Currently there is a recommendation to reduce the consumption of salt due to its relationship with a higher incidence of cardiovascular diseases [3]. This fact is promoting changes from the production industry with the aim of decreasing the use of sodium salt but maintaining the final quality and safety characteristics of the product. In this sense, several strategies, such as the reduction of NaCl addition on the product or its replacement using other salts, have been used even though important changes in the sensory quality and the microbial stability of the final meat products were observed [4].

The reduction of salt results in changes in the complex proteolytic system due to its influence on peptidases which are frequently inhibited in the presence of salt [5,6], leading to unexpected endo- and exopeptidases action and a different final profile of proteolytic products. In this sense, dipeptides are mainly generated by the action of dipeptidyl peptidases (DPPs) from the N-terminal site of proteins and peptides, and peptidyl dipeptidases (DDPs) from the C-terminus [7]. A moderate inhibition of these enzymes was reported during the processing of dry-cured ham due to the action of NaCl. Thus, an increase in the exopeptidase activity would be expected after the reduction of NaCl [7]. These small peptides together with amino acids and some volatile compounds are responsible for the final flavor characteristics observed in dry-cured ham. Furthermore, a wide variety of peptides resulting from the long proteolysis occurred in dry-cured ham have been described to exert certain biological activities [8].

In this regard, dipeptides are of high interest due to their small size that makes them more resistant to enzymes action after ingestion during gastrointestinal digestion, intestinal transport, and circulation in the bloodstream (and therefore more able to reach target organs) [9]. On the other hand, although there is limited information about the effect of salt on the peptide bioactivity, no influence on in vitro angiotensin-I converting enzyme (ACE-I)-inhibitory activity has been reported [10]. Thus, despite the fact that there is some understanding about DPPs and DDPs activity during dry-cured processing, there is very little knowledge about the generated products, especially due to the difficulties in the identification and quantitation of dipeptides in complex salty matrices such as dry-cured ham [11].

The identification of peptides generated during proteolysis of dry-cured ham has been of high interest during the last decade and the obtained results have been used for a better understanding of the phenomena as well as to characterize the potential bioactivity or functional activity that many of these peptides could exert [8]. Up to date, the main methodologies used were based on peptidomic strategies using electrospray ionization (ESI) and matrix-assisted laser desorption/ionization (MALDI) ionization techniques coupled to mass spectrometry in tandem (MS/MS) [12,13]. The data analysis of the obtained spectra for the identification of the peptide sequence was frequently carried out using search engines such as Mascot and protein databases (i.e., UniProt, NCBInr), and a list of peptide sequences as well as their origin proteins were obtained according to a score value. These experimental procedures resulted in the identification of peptides from 4–6 amino acids length as only those peptides with the best score and showing good protein specificity were finally included in the protein score list. However, the identification of dipeptides requires other strategies due to their low abundance and little specificity of their sequence when compared with the theoretical spectra included in the databases.

The strategies for peptide quantitation also depend on the size of the peptides of interest. The most common methodology for the quantitation of peptides is the multiple reaction monitoring (MRM) that is usually developed using a triple quadrupole (QQQ) mass spectrometer, where the ion corresponding to the compound of interest is selected and later fragmented into transitions. Several of these transitions are chosen to obtain the data for quantitation. This approach is very sensitive, accurate, and specific since it permits to selectively quantify compounds within complex samples [14]. However, when analyzing small peptides such as dipeptides, only one transition corresponding to the fragmentation of the amino acids is possible and selected reaction monitoring (SRM) is used as the method of choice [15].

In this study, the dipeptides Pro-Ala (PA), Gly-Ala (GA), Val-Gly (VG), Glu-Glu (EE), Glu-Ser (ES), Asp-Ala (DA), and Asp-Gly (DG) have been identified and quantified in dry-cured ham of 12 months of curing prepared under specific conditions of low-salt content. The role of these dipeptides as bioactive compounds has also been assessed by in vitro inhibition of ACE-I and the inhibition of the pro-inflammatory enzymes neprilysin, tumor necrosis factor-α-converting enzyme (TACE), and autotaxin (ATX). Moreover, molecular docking has been used to predict the ACE-I interactions and the role of the studied dipeptides as taste-related compounds in dry-cured ham has also been discussed.

## 2. Results 

### 2.1. Peptide Quantitation by Tandem Mass Spectrometry

**Figure 1:**
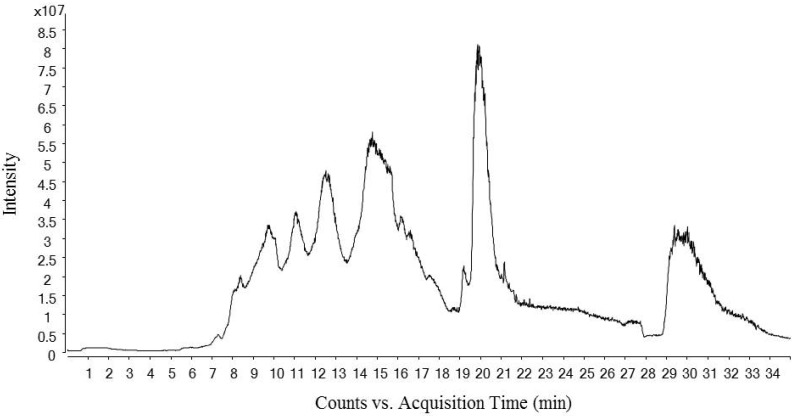
Total Ion Chromatogram (TIC) of a 12 months dry-cured ham extract with low-salt content. The chromatogram represents time (min) in ‘x’ axis versus ions intensity in ‘y’ axis.

**Figure 2:**
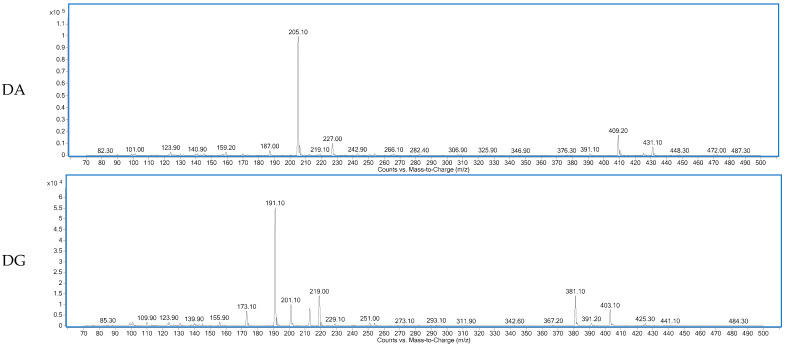

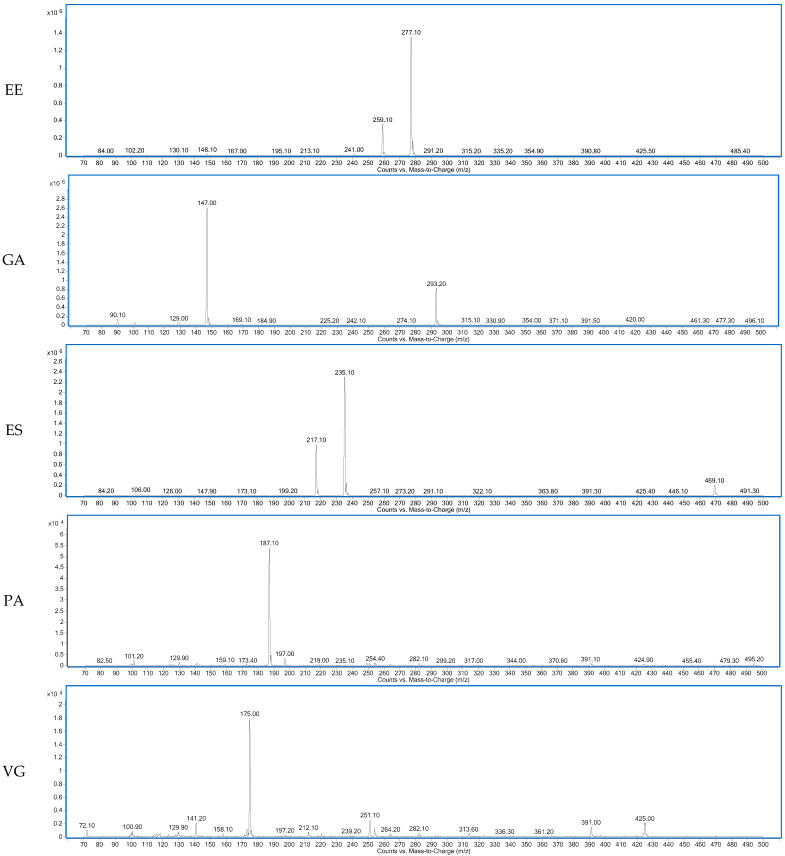
Spectra of the dipeptides DA, DG, EE, GA, ES, PA, VG identified in 12 months dry-cured ham extract with low-salt content.

Figure 1 shows the total ion chromatogram of a dry-cured ham peptides extract analyzed using hydrophilic interaction liquid chromatography (HILIC)-QQQ methodology. The ions were distributed between the minute 7 until the minute 28, when the washing step was established. On the other hand, Figure 2 shows the spectrum of the identified dipeptides where both parent ion and transition are represented. The concentrations of dipeptides were calculated using calibration curves prepared with the corresponding standard (see Supplementary Figure S1) and the quantitative results are shown in Table 1. The highest value was obtained for GA (44.88 μg/g dry-cured ham) and the lowest for PA (0.18 μg/g dry-cured ham), whereas the other peptides values were between 2 and 8 μg/g of dry-cured ham.

### 2.2. ACE-I Inhibitory Activity of the Quantified Dipeptides

#### 2.2.1. In Vitro Results

**Figure 3:**
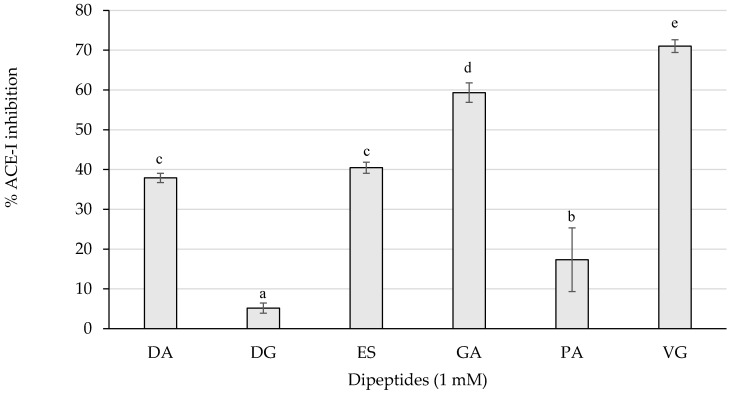
ACE-I inhibition percentages of the dipeptides DA, DG, ES, GA, PA, and VG (EE was null), identified in 12 months dry-cured ham extracts with low-salt content, at 1 mM (*n* > 3). Different letters indicate statistically significant differences (*p* < 0.05) between inhibitory activities.

The ACE-I inhibitory activity of the dipeptides from this study was first screened by measuring the inhibition percentages of ACE-I at 1 mM (Figure 3).

As represented in Figure 3, the dipeptides GA and VG statistically (*p* < 0.05) reached the highest inhibitory activity above 50%, followed by DA, ES, and PA with a weaker influence. Those peptides exerting more than 50% of inhibition were tested for the half maximal inhibitory concentration (IC_50_) value and are shown in Table 2.

#### 2.2.2. Molecular Docking of ACE-I Inhibitory Dipeptides

**Figure 4:**
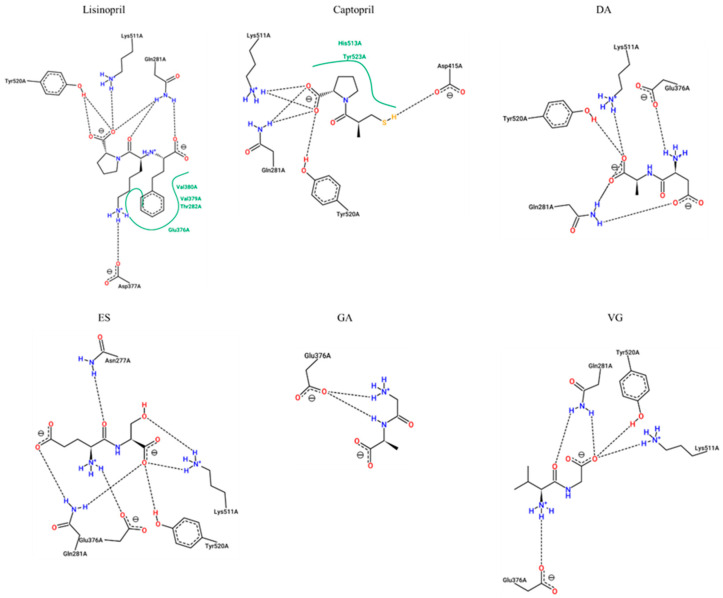
Two-dimensional representation of protein-ligand interactions between ACE-I (ID 1O86) and Lisinopril (PubChem ID: 5362119), Captopril (PubChem ID: 44093), DA (PubChem ID: 5491963), ES (PubChem ID: 6995653), GA (PubChem ID: 1551643), and VG (PubChem ID: 6993111). H bonds are shown as dashed lines, and hydrophobic contacts are represented by green splines; the corresponding pocket residues are shown in the same color. Diagrams were obtained from the ProteinsPlus PoseView tool (access September 2021).

The dipeptides presenting an inhibition percentage greater than 30%, lisinopril, and captopril (positive controls) were studied through molecular docking analysis to understand their potential mechanisms of inhibition. As shown in Table 3 and Figure 4, the estimated interacting residues, binding type, and binding energy of the interactions were calculated.

This data suggested that lisinopril would make interactions with key enzyme residues Asp377, Lys511, His513, Tyr520 and Tyr523, while captopril would be able to associate with His353, Lys511, His513, Tyr520, and Tyr523. These key residues have already been documented for lisinopril and captopril [16,17]. The binding energies are also supported by previous published results, which are very similar [18].

Common interactions were recorded for the dipeptides DA, ES, and VG with enzyme residues His353, Lys511, His513, and Tyr520. On the other hand, GA is suggested to inhibit the enzyme by non-key residues. More interactions with other residues were predicted in all cases, which would contribute to the inhibition. The dipeptide binding affinities are slightly lower than lisinopril and captopril although most of them would show more H-bond interactions. The lowest dipeptide binding affinity was estimated for VG, which may explain a lower IC_50_.

### 2.3. Anti-Inflammatory Activity of the Dipeptides

#### 2.3.1. Neprilysin Inhibitory Activity

**Figure 5:**
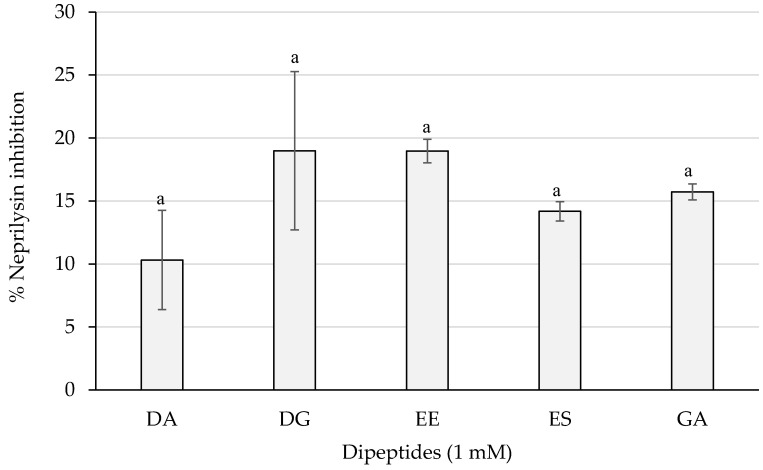
Neprilysin inhibition percentages of the dipeptides DA, DG, EE, ES, and GA (PA and VG were null), identified in 12 months dry-cured ham extracts with low-salt content, at 1 mM (*n* > 3). Similar letters indicate no statistically significant differences (*p* > 0.05).

As shown in Figure 5, dipeptides DG and EE reached the best results with a percentage of almost 20%, whereas PA and VG exerted no inhibition (not shown). However, statistically significant differences were not obtained (*p* > 0.05) between the dipeptide inhibitory activities.

#### 2.3.2. TACE Inhibitory Activity

**Figure 6:**
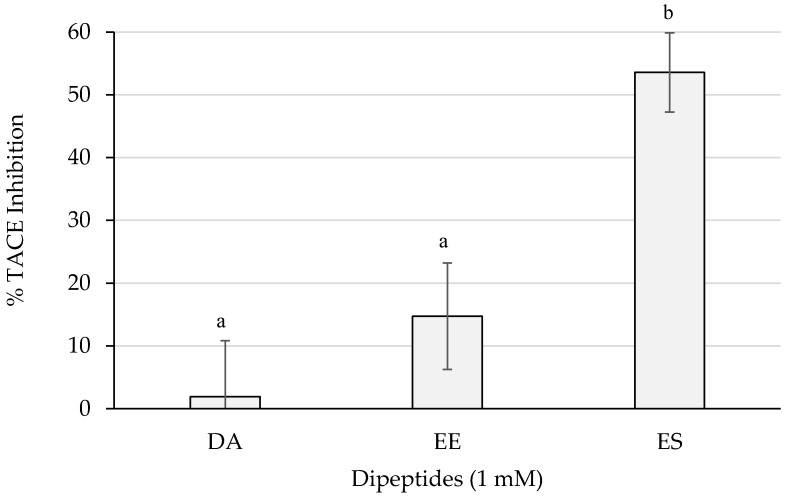
TACE inhibition percentages of the dipeptides DA, EE, and ES (DG, GA, PA, and VG were null), identified in 12 months dry-cured ham extracts with low-salt content, at 1 mM (*n* > 3). Different letters indicate statistically significant differences (*p* < 0.05) between inhibitory activities.

Figure 6 shows the inhibition percentages of TACE. ES reached more than 50% of inhibition which resulted statistically different (*p* < 0.05) from the activities of the rest of the dipeptides. The dipeptide EE surpassed a 10% and DA did not barely reach a 5%.

#### 2.3.3. Autotaxin Inhibitory Activity

**Figure 7:**
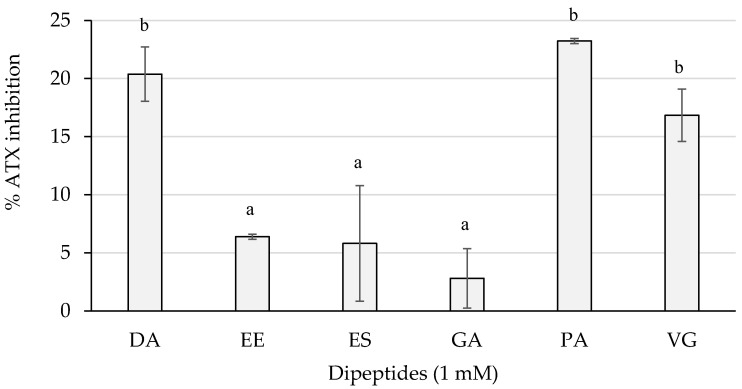
ATX inhibition percentages of the dipeptides DA, EE, ES, GA, PA, and VG (DG was null), identified in 12 months dry-cured ham extracts with low-salt content, at 1 mM (*n* > 3). Different letters indicate statistically significant differences (*p* < 0.05) between inhibitory activities.

Figure 7 shows the inhibition percentages of the dipeptides from this study.

The dipeptides DA and PA reached an average greater than 20%, followed by VG with an inhibition higher than 15%. These three dipeptides presented an inhibition statistically higher (*p* < 0.05) than EE, ES, and GA, whose activity was lower than 10% of inhibition.

## 3. Discussion

### 3.1. Peptide Quantitation by Tandem Mass Spectrometry

The main interests for the identification and quantitation of dipeptides in dry-cured ham are related to: (i) their small size, which allows them to cross the intestinal barrier intact and reach the blood stream. Therefore dipeptides are the best candidates to resist gastrointestinal digestion and intestinal and blood stream transport without being degraded or modified; and (ii) their taste contribution to the development of the dry-cured ham flavor (as the majority of these peptides has been registered in BioPep sensory database) [19]. For instance, dipeptides VG, DA, and DG have been previously described as bitter and umami peptides, whereas PA has showed bitter taste, ES umami and bitterness suppressing, and EE salty, bitterness, and sweetness suppressing capacities.

In this study, HILIC was used to separate a group of dipeptides with different polarity characteristics such as PA, GA, and VG as hydrophobic and ES, EE, DA, and DG with good water solubility. The use of HILIC as a primary separation step instead of the most commonly used reversed-phase has permitted the retention of dipeptides in the HPLC trap column used to concentrate and eliminate the salt during chromatography and before the mass spectrometry (MS) analysis. This approach is cheaper and faster than other procedures used for the removal of salt content before MS analysis, which is an essential step to avoid signal suppression during electrospray ionization [20].

Recently, the content of several dipeptides in traditionally prepared dry-cured ham at different processing times was evaluated. In this study, dipeptides DA, DG, EE, ES, and EV showed an increase in concentration with the time of processing, highlighting the role of endogenous exopeptidases in the generation of dipeptides. Besides, it was found that whereas VG concentration decreased during processing, the dipeptide PA concentration remained constant [21]. The salting step during dry-cured ham processing consists of the addition of salt until reaching a 4–5% of final salt concentration in the product. However, in this study, the final salt concentration was set up to 3.3% in order to establish the effect of salt concentration on the final amounts of the studied dipeptides. Due to the inhibitory effect that salt causes on the activity of endogenous muscle proteases, a higher proteolysis, and hence an increase in the dipeptide concentrations would be expected in low-salted dry-cured hams. Comparing the results obtained in 12 months-aged dry-cured ham samples prepared under traditional conditions and the results obtained in dry-cured hams with a reduced content in salt, no statistical differences were detected except for the dipeptides DA, PA, and VG, whose concentrations were significantly (*p* < 0.05) higher in traditional dry-cured hams (data not shown). These unexpected results could be due to the stability of certain DPPs and DDPs against small changes in salt concentration or also due to an increase of aminopeptidases activity inhibition, which would reduce the hydrolysis rate of the dipeptides and lead to accumulation.

The studied dipeptides have been generated under natural proteolysis conditions mainly due to the action of endogenous muscular DDPs and DPPs. Thus, differences between pigs regarding feeding, slaughter stress, etc., or small differences during ham processing could affect these enzymes behavior and their action during curing could be different between individuals. Thus, high values of standard deviation between samples were expected to be obtained after quantitation by MS due to the high variability between dry-cured ham samples.

Regarding the biological activity of the studied dipeptides, most of their sequences have been previously described in the BIOPEP database as bioactive [22]. Dipeptides GA, VG, DA and DG have been registered as ACE-I inhibitors while PA, GA, VG, and ES have been reported to exert DPP-IV inhibitory activity, which is closely related to the antidiabetic activity. The dipeptide DA has also been reported to reduce the activity of DPP-III in vitro, whereas the dipeptide EE was shown to act as a stimulating vasoactive substance release in human aortic endothelial cells. More recently, dipeptides EE, ES, and DA have been tested for their anticholesterolemic activity, obtaining values of 47.2, 45.5, and 49.6% of HMG-CoA inhibitory activity, respectively, at 1 mM [23]. In this work, the multifunctional properties of the dipeptides were assessed by testing their inhibitory capacity for key enzymes related to cardiovascular and chronic inflammatory pathologies. Such enzymes were ACE-I, neprilysin, TACE, and ATX.

### 3.2. ACE-I Inhibitory Activity of the Quantified Dipeptides

#### 3.2.1. In vitro Results

Several publications have reported the generation of dry-cured ham-derived ACE-I inhibitory peptides. Remarkable sequences were KAAAAP (IC_50_ 19.79 µM), KAAAATP (IC_50_ 25.74 µM), AAPLAP (IC_50_ 14.38 µM), KPVAAP (IC_50_ 12.37 µM), and IAGRP (IC_50_ 25.94 µM); LGL (IC_50_ 145 µM), SFVTT (IC_50_ 395 µM) and GVVPL (IC_50_ 956 µM);AAATP (IC_50_ 100 µM), DVITGA (IC_50_ 900 µM), and ASGPINFT (IC_50_ 975 µM); HCNKKYRSEM, GGVPGG, TKYRVP, TSNRYHSYPWG, and FNMPLTIRITPGSKA, presenting ACE-I inhibitory percentages over 70% at 170 µM [8].

Presumably, longer peptides could give rise to a greater number of interactions, which would be reflected on lower IC_50_ values. However, these are more susceptible of being hydrolyzed in the digestive process, resulting in a significant loss of their inhibitory capacity. The dipeptides assayed in this study showed IC_50_ values similar to those observed in previous publications, which is promising prospect in terms of bioavailability.

#### 3.2.2. Molecular Docking of ACE-I Inhibitory Dipeptides

The outcomes obtained in this paper are in accordance with many peptide-based docking approaches [24]. For instance, peptides LIVT, YLVR, and YLVPH, isolated from pine nut, hazelnut and soy protein, respectively, or Hyp-containing oligopeptides from bovine collagen hydrolysates showed similar binding affinities and also share common enzyme residues [25,26], such as the milk-derived dipeptide GA [27], or the dipeptide LL, identified in a whey hydrolysate, but also generated in dry-cured ham [28,29]. In this line, peptides LGL, SFVTT, and GVVPL identified in dry-cured ham revealed enzyme residues such as Lys511, His513, Tyr520 or Tyr523 [11].

The fact that dipeptides could exert a higher binding affinity may probably be due to a greater capacity to stabilize more interactions and of different nature. However, dipeptides are much less susceptible of being degraded in the assimilation process during digestion than larger peptides. For this reason, the inhibitory potential of short peptides should not be underestimated.

### 3.3. Anti-Inflammatory Activity of the Dipeptides

#### 3.3.1. Neprilysin Inhibitory Activity

Little research on food-derived neprilysin inhibitory peptides exists. Recently, a docking analysis led to the identification of Indonesian herbal candidates for neprilysin inhibitors [30]. It is worth noting that the biologically active B-type natriuretic peptide can be truncated by the enzyme DPP-IV, which is translated in a reduced natriuretic activity. Therefore, it could be argued that it is possible that peptides may be able to regulate degradation of natriuretic peptides via DPP-IV inhibition. In this regard, many DPP-IV inhibitory peptides have been identified in dry-cured ham [8].

To our knowledge, this research reports for the first time the generation of potential neprilysin peptide inhibitors generated during the processing of dry-cured ham. Considering the generation of dry-cured ham-derived ACE-I inhibitory peptides, this data suggests the potential of cardioprotective peptides in dry-cured ham.

#### 3.3.2. TACE Inhibitory Activity

Combinatorial chemical synthesis and subsequent library deconvolution have led to the discovery of in vitro TACE inhibitory peptides, many of them including tripeptides with L-amino acids [31]. Additionally, molecular docking and structure-based rational designs have also been applied to generate a virtual combinatorial library for the further detection of in vitro peptide hydroxamic acid inhibitors (with tripeptide substructure) to target TACE as potential therapeutics for hepatitis [32].

As occurred with the neprilysin enzyme, there is very little research on peptides showing TACE inhibitory activity. However, the present study shows for the first time neprilysin and TACE inhibitory peptides generated in dry-cured ham, which indicates promising prospects to delve into the anti-inflammatory potential of the peptide content of dry-cured ham.

#### 3.3.3. ATX Inhibitory Activity

Recently, thirteen peptides identified in dry-cured showed ATX inhibitory capacities from 5.44% to 57.49%, assayed from stocks at 1 mM (final test concentration of 52.63 µM). The peptide PSNPP was the strongest inhibitor, followed by TGLKP and KAAAATP [33]. Altogether, this data provides information about moderate ATX inhibitory peptides generated in dry-cured ham.

Some of the peptides from this study have exerted more than one inhibitory activity so that the global action may be a benefit for cardiovascular and inflammatory homeostasis. As an example, ACE-I, and PAF-AH are enzymes that are also present in the gut, and recent evidence suggests that inhibition of these enzymes can positively impact gut health and may help to prevent necrosis, ulcerative colitis, and other inflammatory disorders of the human gut [34].

## 4. Materials and Methods

### 4.1. Chemicals and Reagents

The dipeptides PA, GA, VG, EE, ES, and DG were obtained from Bachem AG (Bubendorf, Switzerland), while DA was purchased from Sigma-Aldrich Co. (St. Louis, MO, USA) and used as standards in the optimization of the quantitation methodology.

Angiotensin I-converting enzyme from rabbit lung and captopril were purchased from Sigma-Aldrich (St. Louis, MO, USA), and o-aminobenzoylglycyl-p-nitro-L-phenylalanyl-L-proline (Abz-Gly-p-nitro-Phe-Pro-OH) came from Bachem AG (Bubendorf, Switzerland).

Neprilysin inhibitor screening kit and TACE inhibitor screening assay kit (Catalog number: K996-100 and K366-100, respectively) were purchased by BioVision Inc. (Milpitas, CA, USA). ATX inhibitor screening assay kit (Item No. 700580) was purchased by Cayman Chemical Company (Ann Arbor, MI, USA)

The salt ammonium acetate used on the mobile phase was of MS grade and purchased from Sigma-Aldrich, Co. (St. Louis, MO, USA). H_2_O and acetonitrile (ACN) for mobile phases were of LC-MS grade and purchased from Sharlab, S.L. (Barcelona, Spain). Hydrochloric acid and ethanol were of analytical grade from Sharlab, S.L. (Barcelona, Spain).

### 4.2. Extraction of Peptides and Proteins Precipitation

Six hams were obtained from Landrace × Large White industrial genotypes. The Spanish dry-cured hams were prepared using a traditional protocol in a local factory in Spain with a reduced amount of sodium chloride (final salt content of 3.3%) in comparison with the final concentration of 4–5% sodium chloride that characterize this type of product. The preparation of the dry-cured ham includes (i) a pre-salting step using a mixture of sodium chloride, nitrate and nitrite for 30 min followed by 12 days of salting at 2–4 °C and 90–95% relative humidity; (ii) a post-salting step after the removal of salt excess for 60 days at 4–5 °C and 75–85% relative humidity; and (iii) a curing step up to 12 months of curing at 13–20 °C and 70% relative humidity.

For the study, *Biceps femoris* muscles were excised from the ham pieces and peptides were extracted using 0.01% HCl and later deproteinized by precipitation overnight with ethanol following the methodology of Gallego *et al*., (2015) [35]. The study was carried out with six hams.

### 4.3. Peptides Separation According to the Molecular Weight by Ultrafiltration

In order to separate and concentrate the smaller fraction of generated peptides, a total of 50 mg of each peptide extract was mixed with 1.5 mL of H_2_O and fractionated by ultrafiltration using Amicon^®^ ultra 0.5 mL centrifugal filters (Merk Millipore Ltd., Cork, Ireland) of 3 kDa. The obtained filtrates containing peptides smaller than 3 kDa were freeze-dried. Before the analysis by mass spectrometry in tandem, samples were resuspended in H_2_O up to a final concentration of 10 mg/mL, centrifuged at 4 °C and 10000 g for 10 min, and stored for further analysis by MS. The filtrates were carried out in duplicate.

### 4.4. Identification and Quantitation Using Mass Spectrometry in Tandem

The LC-MS/MS analysis was performed using an Agilent 1260 Infinity LC system (Agilent, Palo Alto, CA, USA) coupled to a QQQ 6420 Triple Quad LC/MS (Agilent, CA, USA) with an ESI.

A total of 5 µL of each sample was injected and concentrated on a SeQuant ZIC^®^–HILIC guard fitting PEEK column (5 µm, 14 mm × 1 mm; Merk KGaA, Darmstadt, Germany) at a flow rate of 0.02 mL/min for 5 min and using 90% (*v*/*v*) ACN in 10 mM ammonium acetate as mobile phase. The trap column was automatically switched in-line onto a SeQuant ZIC^®^–HILIC capillary column (5 µm, 150 mm × 0.3 mm; Merk KGaA, Darmstadt, Germany). Mobile phases were 10 mM ammonium acetate as solvent A, and ACN as solvent B. Gradient elution for LC was 0–8 min, 80% B; 8–25 min, linear from 80 to 30% B; 25–28 min 30% B; and 28–35 min, linear from 30 to 80% B; at a flow rate of 6 μL/min at 30 °C. The column outlet was directly coupled to an ESI, and the QQQ (MS/MS) was operated in positive polarity to acquire full scan mass spectra from 70 to 500 m/z. Other MS parameters were: nitrogen gas flow, 6 L/min; gas temperature, 350 °C; nebulizer pressure, 15 psi; capillary, 3500 V; fragmentor, 100 V; scan time, 500 ms; cell accelerator, 4 V.

The standards were prepared at a concentration of 1 nmol/µL and analyzed using previously described methodology to get their m/z ratio and their specific retention times. The data obtained from the standard peptides was used to confirm the presence of the dipeptides in dry-cured ham extracts, and different calibration curves according to the observed peak areas obtained from extracted ion chromatograms (XICs) in samples were prepared. The effect of the matrix and possible interferences were evaluated by spiking the sample with the dipeptide standards, and the spectra and retention time were confirmed. The analysis of the samples was carried out using MassHunter LC/MS Data Acquisition (version B.08.00) and the data analysis of the obtained results was carried out using MassHunter Qualitative Analysis software (version B.07.00) (Agilent Technologies, Inc.). The analysis of the standard dipeptides and dry-cured ham samples (*n* = 6) were carried out in triplicate.

### 4.5. ACE-I Inhibitory Activity of the Identified/Quantified Dipeptides

#### 4.5.1. In vitro Assay

The same methodology described by Sentandreu and Toldrá [36] was followed in order to test the ACE-I inhibitory capacity of the dipeptides. The assay is based on the fluorometric measurement of the increase in fluorescence, which represents the enzymatic hydrolysis of the substrate by ACE-I in presence of the test compound.

Each reaction was prepared by adding the reagents in the following order: 50 µL of sample were mixed with 50 μL of enzyme (3 mU/mL ACE-I in 150 mM Tris base buffer, pH 8.3), and the reaction was initiated by adding 200 μL of substrate (0.45 mM Abz-Gly-p-nitro-Phe-Pro-OH in 150 mM Tris base buffer with 1.125 mM NaCl, pH 8.3). Dipeptide solutions at different concentrations (to a final concentration of 0.01, 0.1, 0.25, 0.5, 1, 2, 5 and 10 mM), bidistilled water (100% activity) and captopril (reaching a final concentration of 10 μM; positive control) were tested. Immediately, the components were mixed, and the fluorescence at 355 nm excitation and 405 nm emission wavelengths was monitored from 0 to 45 min at 37 °C by a Fluoroskan Ascent FL (Thermo Electron Corporation Labsystems, Helsinki, Finland).

The Abz-Gly-p-nitro-Phe-Pro-OH dependent hydrolysis of ACE-I in the absence (100% activity) and presence of a dipeptide or captopril was measured by the fluorescence gain, which is directly proportional to enzyme activity. Then, enzyme inhibition was calculated as a percentage yield considering the fluorescence increase in the sample with respect to 100% activity control. Finally, the IC_50_ values for the most inhibitory dipeptides were determined by regression analysis of the inhibitory activity versus sample concentration. All reactions were assayed in triplicate.

#### 4.5.2. Molecular Docking with ACE-I

Based on the inhibitory results, the dipeptides stimulated the interest for a further in silico analysis in order to predict its potential interacting mechanism with the enzyme. The dipeptide sequences (PubChem ID: 5491963, 6995653, 1551643 and 6993111 for DA, ES, GA and VG, respectively), as well as those from captopril (PubChem ID: 44093) and lisinopril (PubChem ID: 5362119), were obtained in “sdf” format from PubChem [37] and the pdb files were extracted using Discovery Studio Visualizer v20.1.0. 19295 (Dassault Systèmes BIOVIA Corp., 2020). The structure of human C-domain ACE-I (protein data bank ID: 1O86), in complex with lisinopril [38], was downloaded from Protein Databank (PDB) [39].

Human ACE-I (EC 3.4.15.1) consists of two functional domains, namely N and C. Interestingly, targeting the C domain was found to be sufficient for controlling blood pressure, and therefore, all inhibitors target this site [25]. Three main active site pockets have been reported: S1 (Ala354, Glu384 and Tyr523), S2 (Gln281, His353, Lys511, His513 and Tyr520) and S1′ (Glu162) [40]. Within the active sites of both domains, there is located a zinc-binding motif with two histidine residues (His383 and His387) coordinated with a zinc ion (+2) [40].

Ligand-protein docking simulations were carried out using AutoDock tools v1.5.6 and AutoDock v4.2.5.1 (The Scripps Research Institute) programs [41,42]. Gasteiger charges and hydrogens were added to all molecules, water molecules and original lisinopril were also removed from the enzyme, and ligand torsions were detected by AutoDock. Structure data files were converted into the Protein Data Bank partial charge and atom type format.

Firstly, it was accomplished a preliminary test to obtain more information about the coordinates of the area for screening. Insights were made by submitting the 1O86 pdb archive to ProteinsPlus (https://proteins.plus/, accessed on 15 September 2021), and processing the molecule with the tools PoseView [43,44] and DoGSiteScorer [45] (https://proteins.plus/, accessed on 15 September 2021). Additionally, the FASTA archive of the enzyme was analyzed with the tool InterPro database [46] (https://www.ebi.ac.uk/interpro/, accessed on 15 September 2021) for the prediction of the active site.

The definitive Grid Box (70 × 70 × 60) was centered on one of the ACE-I and lisinopril binding site where active residues locate, with coordinates X = 43.946, Y = 40.191, and Z = 33.879 [47], and spacing of 0.375 Å. Fifty docking runs were performed, using a Lamarckian genetic algorithm between flexible ligand and rigid receptor, a population size of 150, a maximum of 2,500,000 generations and 2,500,000 evaluations for 50 GA runs. The root means square deviation tolerance was set to 2.0 Å for the clustering of docking results. Analysis of the results was carried out by sorting the different complexes with respect to the predicted binding energy. The pose with lowest binding energy in each case was individually examined, and interactions were processed with online software Protein-Ligand Interaction Profiler (PLIP) [48] (https://plip-tool.biotec.tu-dresden.de/plip-web/plip/index, accessed on 20 September 2021), to validate the interactions; and with ProteinsPlus, to obtain the two-dimensional representations by using PoseView algorithm [49].

### 4.6. Anti-Inflammatory Activity of the Dipeptides

#### 4.6.1. Neprilysin Inhibitory Screening Assay

The assay monitors the cleavage of the fluorescent group 4-methylcoumaryl-7-amide from a peptide substrate by neprilysin. The fluorescent signal is inversely proportional to inhibition. The dipeptides were assayed at 1 mM according to the manufacturer instructions by triplicate. Briefly, dipeptides were dissolved in assay buffer at a concentration of 10 mM, and 10 µL were used for the assay. Parallelly, 10 µL of 1:100 diluted thiorphan (positive control) and 10 µL of assay buffer (100% activity control) were also tested. A similar volume of neprilysin working solution (previously diluted 1:10 in assay buffer) was mixed with the test compounds and controls. Suddenly, well volume was adjusted up to 80 µL. Then, the mixtures were incubated at 37 °C for 10 min in dark. Finally, reactions were triggered by the addition of 20 µL of substrate working solution (previously diluted 1:40 in assay buffer) to each well. Fluorescence was monitored (ex/em = 320/420 nm) in kinetic mode at 37 °C for 60 min. The slopes of the linear range of the plots fluorescence vs time were considered for the relative inhibition calculations, taking into account the 100% activity.

#### 4.6.2. TACE Inhibitory Screening Assay

By the measure of fluorescence, the hydrolysis of a FRET substrate by TACE was examined. The signal is also inversely proportional to the activity of an inhibitor.

Peptide triplicates at 1 mM were proved conforming to the protocol provided along with the kit. Indeed, 25 µL of 4 mM peptide solutions dissolved in assay buffer, 25 µL of 1:24 diluted in assay buffer GM6001 (positive control) and 25 µL of assay buffer (100% activity control) were mixed with 50 µL of TACE solution, previously diluted 1:25 with assay buffer. After incubation at 37 °C for 5 min, a volume of 25 µL of substrate solution (previously diluted 2:25 in assay buffer) was added to the reaction mixtures. The reactions were monitored by measurement of the fluorescence at ex/em 318/449 nm, under 37 °C for 30 min. The relative fluorescence values were used for relative inhibition calculation, considering the 100% activity.

#### 4.6.3. ATX Inhibitory Screening Assay

In this assay, ATX cleaves bis-(p-nitrophenyl) phosphate liberating p-nitrophenol, a yellow product that is measured at 405–415 nm. Hence, the absorbance is inversely proportional to inhibitory activity. The dipeptides at 1 mM were assayed by triplicates using the protocol’s steps provided with the kit. Prior steps were carried out for reagent preparation. The assay buffer was diluted 1:10 with bidistilled water. Using this diluted assay buffer, the ATX assay reagent was 1:10 diluted, and the substrate reconstituted. Additionally, the HA-155 positive control was diluted in assay buffer to reach a final concentration in the assay well of 1 µM. Then, 10 µL of each dipeptide solution, positive control working solution, and diluted buffer assay (100% initial activity control), were mixed with 10 µL of ATX working solution and 150 µL of assay buffer. A background control was included by mixing 160 µL of assay buffer with 10 µL of assay buffer. Suddenly, 20 µL of the substrate working solution were added to each well. The reactions were incubated at 37 °C for 30 min and finally, the absorbance was measured at 405 nm. After subtracting the background absorbance from the peptide, positive and 100% initial activity controls absorbance values, the inhibition percentage was calculated as a rate in respect with the 100% initial activity.

### 4.7. Statistical Analysis

One-way analysis of variance (ANOVA) and multiple comparisons of means by Tukey contrasts tests were performed using R software (R Foundation for Statistical Computing, Vienna, Austria), to determine statistical differences between inhibition percentages of the dipeptides. Results were expressed as the mean of three replicates ± standard deviations.

## 5. Conclusions

The quantitation of dipeptides PA, GA, VG, EE, ES, DA, and DG in low-salted Spanish dry-cured ham using a triple quadrupole mass spectrometry in tandem instrument demonstrates the advantages of the implementation of hydrophilic interaction liquid chromatography in the removal of salt and the concentration of dipeptides in complex food matrices, in order to avoid ion suppression during the analysis and increase the abundance of the dipeptides of interest. The dipeptides successfully quantitated were found in concentrations ranging from 0.18 to 44.88 µg/g of dry-cured ham. Although reduction of salt did not affect the concentration of most of the dipeptides, DA, PA, and VG showed a significantly lower concentration in low-salt dry-cured ham in comparison with the traditional product.

The obtained results elucidate the importance of the generation of dipeptides by the action of endogenous enzymes during curing, especially in low-salt content ham where the enzyme activity is higher. The possibility of quantifying such dipeptides is very important to estimate their contribution to umami and bitter tastes but also to evaluate their potential as bioactives and, therefore, to contribute to better consumer’ health. In this regard, the inhibitory capacity of the studied dipeptides proved the intense generation of potential cardioprotective-related multifunctional peptides in dry-cured ham (particularly via ACE-I inhibition). The higher probability of short peptides to exert a bioactive effect in comparison with longer peptides makes dry-cured ham a valuable potential source of such compounds.

## Figures and Tables

**Table 1:** Dipeptides identified and quantified using mass spectrometry in tandem in a triple quadrupole instrument.

Peptide	Sequence	MW ^1^	t_R_ ^2^	Amount ^3^	SD ^4^
PA	Pro-Ala	186.21	16.2	0.18	0.13
GA	Gly-Ala	146.15	15.6	44.88	22.27
VG	Val-Gly	174.2	12.5	2.11	1.80
EE	Glu-Glu	276.24	17.2	8.42	3.03
ES	Glu-Ser	234.21	16	4.43	1.28
DA	Asp-Ala	204.18	15.7	7.82	1.83
DG	Asp-Gly	190.15	15.9	8.28	1.83

^1^ Molecular weight; ^2^ time of retention according to the total ion chromatogram; ^3^ concentration (µg/g) in 12 months dry-cured ham low in salt; ^4^ standard deviation.

**Table 2:** IC_50_ values of the most ACE-I inhibitory dipeptides from the study.

Sequence		IC_50_ Value
		(µM)
GA	Gly-Ala	516.879
VG	Val-Gly	377.669

**Table 3:** ACE-I binding site residues involved in docking interactions with lisinopril, captopril and dipeptides, with docking scores.

Ligand	Binding Energy (kcal/mol)	Inhibition Constant (µM)	Protein Residues Involved in H-Bond Interactions [Chain: Residue (Distance Btw Donor-Acceptor) (Protein Donor/Acceptor, Residue From Side Chain)]	Protein Residues Involvedin Hydrophobic Interactions [Chain: Residue (Distance Btw Carbon Atoms)]	Protein Residues Involved in Salt Bridges [Chain: Residue (Distance Btw Centers Of Charge) (Ligand Functional Group Providing the Charge)]
Lisinopril	−6.08	35.21	A:Gln281 (2.96 Å) (Donor,sd) A:Asp377 (2.77 Å) (Acceptor,sd) A:Lys511 (3.50 Å) (Donor,sd) A:Tyr520 (2.71 Å) (Donor,sd)	A:Thr282 (3.49 Å) A:Glu376 (3.20 Å) A:Val379 (3.30 Å) A:Val380 (3.66 Å) A:Tyr523 (3.69 Å)	A:Lys511 (3.66 Å) (Carboxilate) A:His513 (3.55 Å) (Carboxilate)
Captopril	−5.96	42.91	A:Gln281 (3.28 Å) (Donor,sd) A:**Tyr520** (2.91 Å) (Donor,sd)	A:Phe457 (3.15 Å) A:**Tyr523** (3.89 Å) A:Phe527 (3.04 Å)	A:**His353** (5.05 Å) (Carboxilate) A:**Lys511** (2.57 Å) (Carboxilate) A:**His513** (4.54 Å) (Carboxilate)
DA	−4.88	263.8	A:Gln281 (3.09 Å) (Donor,sd) A:Glu376 (2.62 Å) (Donor,sd) A:Glu376 (2.62 Å) (Acceptor,sd) A:**Tyr520** (3.26 Å) (Donor,sd)	A:Thr282 (3.96 Å)	A:**His353** (5.03 Å) (Carboxilate) A:**Lys511** (2.48 Å) (Carboxilate) A:**His513** (4.84 Å) (Carboxilate)
ES	−4.50	499.08	A:Asn277 (2.96 Å) (Donor,sd) A:Gln281 (3.00 Å) (Donor,sd) A:Thr282 (3.34 Å) (Donor,sd) A:Glu376 (2.47 Å) (Donor,sd) A:Glu376 (2.47 Å) (Acceptor,sd) A:**Lys511** (3.02 Å) (Donor,sd) A:**Tyr520** (3.05 Å) (Donor,sd)	A:Thr282 (3.91 Å)	A:**His353** (5.40 Å) (Carboxilate) A:**Lys511** (3.68 Å) (Carboxilate) A:**His513** (4.84 Å) (Carboxilate)
GA	−5.06	196.38	A:Asn277 (3.43 Å) (Donor,sd) A:Gln281 (3.35 Å) (Donor,sd) A:Thr282 (3.93 Å) (Donor,sd) A:Glu376 (2.84 Å) (Donor,sd) A:Glu376 (2.41 Å) (Acceptor,sd) A:Glu376 (2.84 Å) (Acceptor,sd) A:Glu376 (3.25 Å) (Donor)	A:Thr282 (3.59 Å)	*Absent*
VG	−5.10	183.45	A:Asn277 (3.92 Å) (Donor,sd) A:Gln281 (2.97 Å) (Donor,sd) A:Thr282 (3.99 Å) (Donor,sd) A:Thr282 (3.99 Å) (Acceptor,sd) A:Glu376 (2.49 Å) (Donor,sd) A:**Tyr520** (3.17 Å) (Donor,sd)	A:Thr282 (3.52 Å) A:Glu376 (3.52 Å) A:Val380 (3.43 Å)	A:**His353** (4.87 Å) (Carboxilate) A:**Lys511** (2.79 Å) (Carboxilate) A:**His513** (4.78 Å) (Carboxilate)

Key residues of the binding site are highlighted in bold. Common residues whereby pravastatin and dipeptides interact with are underlined.

## Data Availability

Data is contained within the article. The data presented in this study is available in the present article.

## Supplementary Materials

The following supporting information can be downloaded at: https://www.mdpi.com/article/10.3390/ijms23052507/s1.
