# Supplementary material for: Identification and Quantitation of Bioactive and Taste-Related Dipeptides in Low-Salt Dry-Cured Ham
Source: Int J Mol Sci. 2022 Feb 24;23(5):2507. doi: 10.3390/ijms23052507 (PMC8910418; doi:10.3390/ijms23052507)
Supplement: Supplementary file 1 [file ijms-23-02507-s001.zip › ijms-1611150-supplementary.pdf]

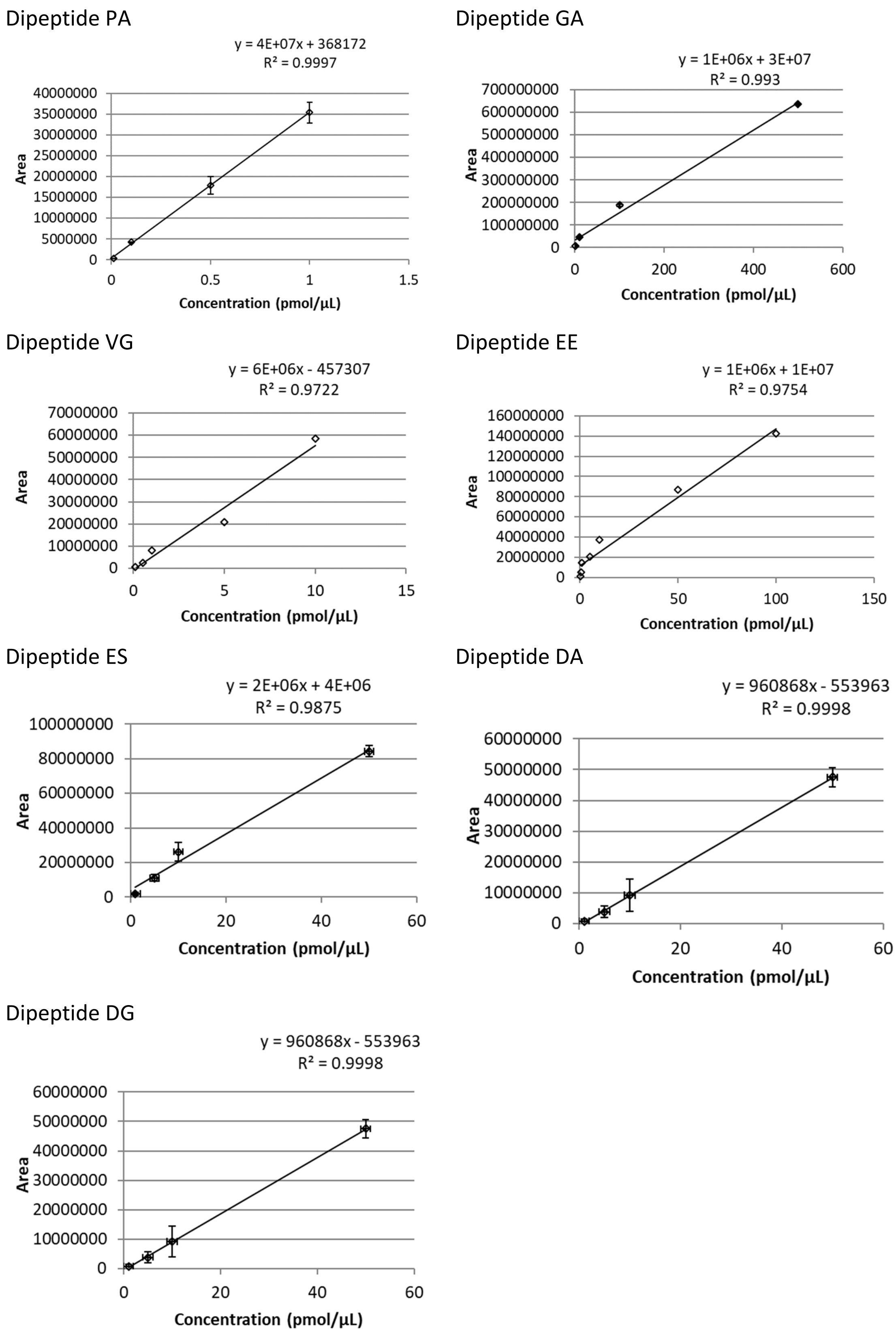

Supplementary Figure S1. Calibration curves and regression model used in the quantitatioon of the dipeptides.
